# Plk1 Regulates the Repressor Function of FoxM1b by inhibiting its Interaction with the Retinoblastoma Protein

**DOI:** 10.1038/srep46017

**Published:** 2017-04-07

**Authors:** Nishit K. Mukhopadhyay, Vaibhav Chand, Akshay Pandey, Dragana Kopanja, Janai R. Carr, Yi-Ju Chen, Xiubei Liao, Pradip Raychaudhuri

**Affiliations:** 1 Department of Biochemistry and Molecular Genetics (M/C 669), University of Illinois, College of Medicine, 900 S, USA Ashland Ave., Chicago, IL-60607, USA; 2 Department of Hematology/Oncology, University of California, Los Angeles, CA, USA; 3 Abramson Family Cancer Research Institute, University of Pennsylvania, Philadelphia, PA 19104, USA; 4 Jesse Brown VA Medical Center, 820 S. Damen Ave., Chicago, IL-60612, USA

## Abstract

FoxM1b is a cell cycle-regulated transcription factor, whose over-expression is a marker for poor outcome in cancers. Its transcriptional activation function requires phosphorylation by Cdk1 or Cdk2 that primes FoxM1b for phosphorylation by Plk1, which triggers association with the co-activator CBP. FoxM1b also possesses transcriptional repression function. It represses the mammary differentiation gene GATA3 involving DNMT3b and Rb. We investigated what determines the two distinct functions of FoxM1b: activation and repression. We show that Rb binds to the C-terminal activation domain of FoxM1b. Analyses with phospho-defective and phospho-mimetic mutants of FoxM1b identified a critical role of the Plk1 phosphorylation sites in regulating the binding of FoxM1b to Rb and DNMT3b. That is opposite of what was seen for the interaction of FoxM1b with CBP. We show that, in addition to GATA3, FoxM1b also represses the mammary luminal differentiation marker FoxA1 by promoter-methylation, and that is regulated by the Plk1 phosphorylation sites in FoxM1b. Our results show that the Plk1 phosphorylation sites in FoxM1b serve as a regulator for its repressor function, and they provide insights into how FoxM1b inhibits differentiation genes and activates proliferation genes during cancer progression.

The transcription factor FoxM1 is a driver of cancer progression. Its over-expression coincides with high-grade progression in almost all types of cancers, including those in breast^1^,^2^, liver^3^,^4^, lung^5^,^6^, prostate^7^,^8^, pancreas^9^,^10^, ovary^11^, brain^12^, nasopharynx^13^, esophagus^14^, and also in certain leukemia^15^. Moreover, a study that analyzed 18,000 human tumors with outcomes for 39 different malignancies identified over-expression of FoxM1 as a major predictor for poor prognosis^16^. FoxM1 is critical for the cancer signaling pathways. For example, activated H-Ras in liver increases expression of FoxM1, which is important for progression of H-Ras-induced hepatocellular carcinoma^4^. Deletion of FoxM1 after the development of hepatocellular carcinoma inhibits progression. In breast cancers, FoxM1 has been shown to form a positive feedback loop with the PDGF/Akt signaling pathways^17^. Also, it promotes TGF-b signaling through sustained activation of SMAD3/SMAD4^18^. In glioblastoma, as well as in pancreatic cancer FoxM1 is critical for Wnt/b-catenin signaling^19^. FoxM1 was shown to participate in the nuclear accumulation of b-catenin following activation of Wnt-signaling. Moreover, in glioblastoma, it promotes nuclear accumulation of GLI1, a mediator of the Hedgehog pathway, by increasing expression of Importin-7^20^. FoxM1 has been linked also to STAT3 activation in gliobastoma^21^. A recent study on soft-tissue sarcoma also identified FoxM1 downstream of the Hippo signaling pathway^22^. FoxM1 is also a target of the tumor suppression pathways. For example, p53 is a potent suppressor of FoxM1 expression^23^,^24^. P19Arf associates with FoxM1 and re-localizes it to the nucleolus^25^. Also, vitamin D signaling in pancreatic cancer suppresses progression by inhibiting expression of FoxM1^26^.

FoxM1 is a pro-proliferation transcription factor. It stimulates expression of genes important for G1 to S progression, as well as mitotic progression. FoxM1 directly binds to its cognate element in the promoter of cell cycle genes and stimulates their expression^27^,^28^. B-Myb and the MuvB complex have been shown to facilitate recruitment of FoxM1 onto the promoters of several mitotic genes^29^. FoxM1 is involved also in the activation of anti-oxidant genes. For example, FoxM1 directly stimulates expression of Catalase, MnSOD and PRDX3^30^. The anti-oxidant function of FoxM1 is particularly important for cancer cells that express reactive oxygen species (ROS) producing oncogenes, such as activated Ras and Akt. In pancreatic cancer cells FoxM1 participates in Warburg effect by stimulating expression of LDHA^31^. FoxM1 also stimulates expression of several genes involved in invasion, pre-metastatic niche formation and other steps involved in metastasis^32^. FoxM1 also induces expression of genes that drive epithelial to mesenchymal transition (EMT)^12^,^33^,^34^,^35^,^36^. Moreover, FoxM1 activates expression of several reprogramming genes, including c-Myc, Sox2, Nanog, Oct4, and BMI1^4^,^37^,^38^. Consequently, FoxM1 is important for cancer stem cells, depletion of FoxM1 leads to a loss of the cancer stem cells^4^. These are consistent also with the observations that over-expression of FoxM1 leads to drug resistance^39^,^40^,^41^.

FoxM1 is cell cycle-regulated. The transcriptional stimulatory function of FoxM1 requires its activation by the Cyclin/Cdk kinase and Plk1^42^,^43^. The Cdk1/Cdk2 kinases phosphorylate FoxM1 (isoform b) at the T596 residue that serves as a primer for phosphorylation by the Plk1 kinase at residues S715 and S724 of FoxM1. A phospho-mimetic mutant at the two Plk1 phosphorylation sites was shown to be constitutively active in stimulation of transcription^42^. Because those kinases are cell cycle regulated, the transcriptional stimulatory activity of FoxM1 increases reaching maximal stimulatory activity in G2 and early M phases, where Cdk1 is maximally active and Plk1 is expressed at high levels by FoxM1^42^,^43^. It is noteworthy that FoxM1 stimulates expression of Cdc25b and Cyclin B1 that activate Cdk1^44^. Thus, FoxM1, Cdk1/Cdk2 and Plk1 activate each other by feedforward mechanisms in G2/M phases. At the end of M phase, FoxM1 becomes de-phosphorylated, and in early G1 phase it is degraded by the Ub-proteasome pathway involving APC/C-Cdh1^45^,^46^. Recent studies also indicated that Cdk4/Cdk6 stabilize FoxM1 by phosphorylation at multiple sites^47^.

FoxM1 also possesses transcriptional repression function that is significant in regulating differentiation. In mammary gland FoxM1 is expressed at high levels in the luminal progenitor cells, and it represses expression of the luminal differentiation factor GATA3^1^. Deletion of FoxM1 increases expression of GATA3 and decreases the pool of the mammary stem and luminal progenitor cells. The opposite is true when FoxM1 is over-expressed in the mammary gland^1^. The repression of GATA3 by over-expressed FoxM1 requires Rb. Depletion of Rb attenuates the FoxM1 mediated increase in the stem/progenitor cells^1^. Interestingly, the GATA3 repression function of FoxM1 is detected also in human breast cancer cell lines^1^. We showed that FoxM1 represses expression of GATA3 by inducing methylation at the CpG islands in the GATA3 promoter by recruiting DNMT3b. Moreover, FoxM1-driven methylation of the GATA3 promoter involves Rb^1^. In this study we describe a new function of Plk1 in regulating the repressor/methylation activities of FoxM1 by controlling its interaction with Rb and DNMT3b.

## Results

### Rb and DNMT3b associate with FoxM1b involving distinct domains

**Figure 1:**
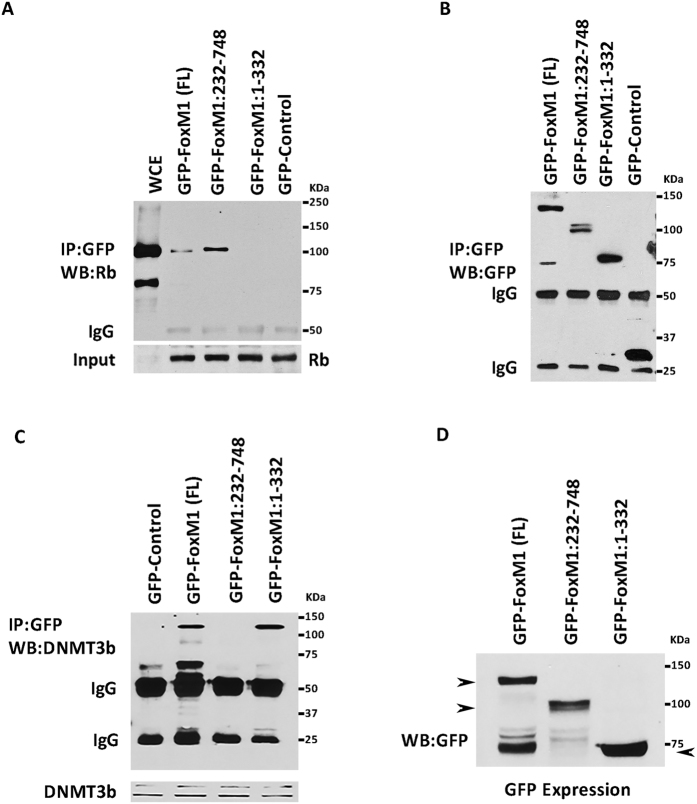
Rb and DNMT3b bind to different regions in FoxM1b. MCF7 cells were transiently transfected with GFP-Control, GFP-FoxM1 full length (FL), GFP-FoxM1 mutant (232–748) or a GFP fusion construct containing the N-terminal 331 residues of FoxM1. 48hr post transfection, lysates (800 ug) were immunoprecipitated with GFP antibody and fractionated by SDS PAGE. Western blot analysis was performed with a monoclonal antibody specific for Rb (**A**) or antibody against DNMT3b (**C**). Immunoprecipitations of the GFP-fusion proteins are shown in panel B, and expression of GFP constructs in experiment C are shown in panel D.

**Figure 2:**
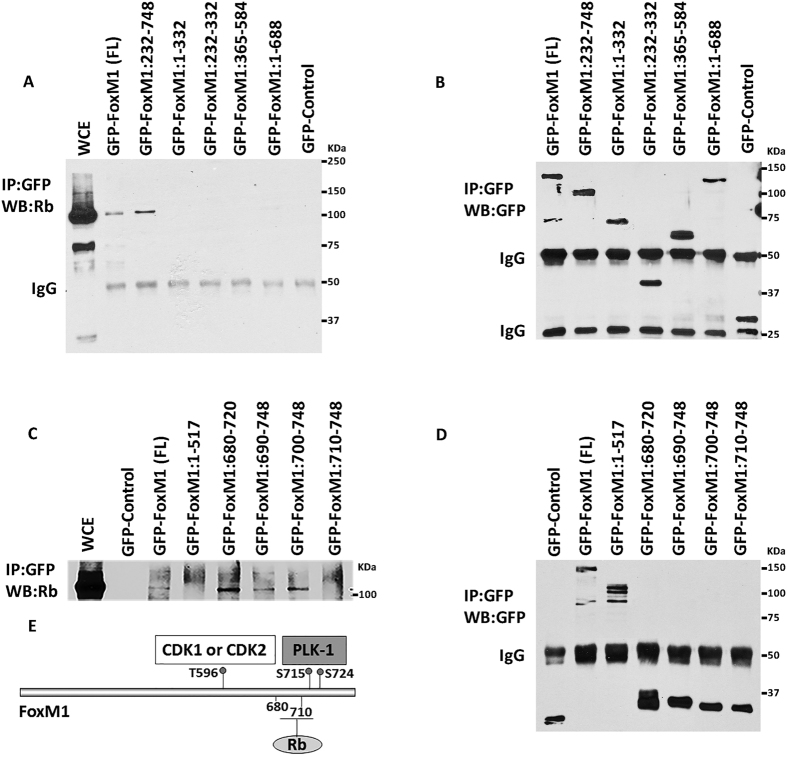
Rb binding site overlaps with the Plk1 phosphorylation sites in FoxM1b. MCF7 cells were transiently transfected with GFP-FoxM1 (FL) or the indicated FoxM1 deletion mutants. Cell lysates were prepared from transfected cells after 48 hr. Cell lysates (700 ug) were immunoprecipitated with GFP antibody followed by western blotting with Rb-antibody (**A,C**). Immunoprecipitation of the GFP constructs are shown in panels B and D. (**E**) Schematic representation of the Rb binding site within the C-terminal domain of FoxM1.

Several studies including ours demonstrated that FoxM1 binds to Rb^48^,^49^. Recently, we showed that FoxM1 interacts with the *de novo* DNA-methyltransferase DNMT3b^1^. In that study, we showed that Rb also binds to DNMT3b, and that Rb is critical for the FoxM1-DNMT3b mediated methylation of the CpGs in the GATA3 promoter. Therefore, we investigated whether FoxM1 interacts with DNMT3b indirectly through its interaction with Rb. We analyzed mutants of FoxM1b for interactions with Rb and DNMT3b to see whether the interaction with DNMT3b depends upon the Rb binding site in FoxM1b. GFP-fusion constructs of FoxM1b mutants were expressed in MCF7 cells to investigate the interactions using co-immunoprecipitation experiments. Interestingly, we observed that an N-terminal deletion mutant lacking residues 1–232 could associate with Rb as efficiently as the wild type FoxM1 (Fig. 1A,B), however, that mutant failed to bind DNMT3b (Fig. 1C,D). On the other hand, a mutant of FoxM1 containing the N-terminal residues 1–332 was able to associate with DNMT3b but not with Rb (Fig. 1A,C). This N-terminal region in FoxM1 is distinct from the Rb-binding domain identified previously. A previous study^49^, using GST pull-down assays, identified a central domain in the FoxM1c isoform, distinct from the N-terminal region that binds DNMT3b, which binds Rb. To further investigate that region in FoxM1b for Rb-binding, we analyzed additional GFP-FoxM1b mutants. Surprisingly our co-immunoprecipitation experiments identified a different region in the C-terminus of FoxM1b as the Rb-binding site. A mutant of FoxM1b (1–688) lacking the C-terminal 80 residues failed to bind Rb (Fig. 2A,B). On the other hand, a GFP-fusion protein containing residues between 680 and 720, which is critical for transactivation function of FoxM1b, could efficiently bind Rb (Fig. 2C,D). Based on our binding data and secondary structure prediction analyses using the Robetta program (WWW.robetta.org), we think that the Rb-binding site lies between residues 700 and 722 of FoxM1. The sequence L (707)VLDTMNDSLSKILLD(722) of FoxM1 has typical amino acid sequence pattern of a helix. Deletion of residues L(707)VL disrupts the helical structure of this sequence and, therefore, predicted to disrupt the interaction with Rb. We suspect, that is why there is a loss of binding with our construct GFP-FoxM1:710–748. Thus, while DNMT3b binding involves the N-terminal region in FoxM1b, Rb binding involves the C-terminal activation domain in FoxM1b. Expression and immunoprecipitation data for the GFP-FoxM1b proteins are included also in supplemental Fig. S1.

### Rb associates with FoxM1b lacking Plk1 phosphorylation

**Figure 3:**
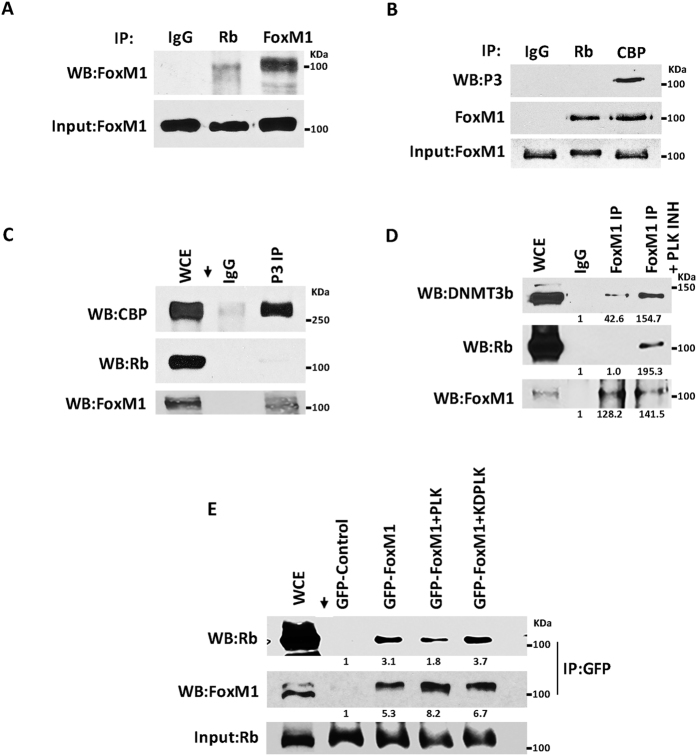
Plk1 phosphorylation of FoxM1b regulates its interaction with Rb. (**A**) Rb interacts with an underphosphorylated form of FoxM1. Cell extracts prepared from MCF7 cells were immunoprecipitated with IgG, Rb, and FoxM1 antibody. Immunoprecipitates were subjected to western blot analysis with pan FoxM1 antibody. (**B**) A phospho-specific antibody (P3) was generated using a phospho-peptide corresponding to the Plk1-sites in FoxM1 and this antibody detects FoxM1 in CBP immunoprecipitate. Extracts of MCF7 cells were immunopreciptated with IgG, Rb or CBP antibody and subjected to western blotting that was probed with the P3 antibody or pan-FoxM1 antibody. (**C**) Extracts of MCF7 cells were immunoprecipitated with either IgG or the P3-ab. The immunoprecipitates were analyzed by western blotting with CBP-ab or Rb-ab. (**D**) MCF7 cells were treated with a Plk1 inhibitor (BI 2536) for 24 h at 100 nM concentration or with DMSO. Cell extracts were immunoprecipitated with either IgG or FoxM1-antibody and western blotted for FoxM1, Rb and DNMT3b. (**E**) Exogenous Plk1 kinase expression inhibits Rb binding to FoxM1. Wild type FoxM1 was expressed in MCF7 cells together with either kinase active PLK (PLK) or kinase dead PLK (KDPLK). Extracts were immunopreciptated with GFP antibody and western blotted for Rb.

Previous studies showed that FoxM1b binds to under-phosphorylated Rb in G1 phase^48^. Because the Rb-binding region in FoxM1b partly overlaps with the Plk phosphorylation sites (S715 and S724), we investigated how Plk1-phosphorylation of FoxM1b would affect the interaction with Rb. First, we immunoprecipitated Rb and FoxM1 and analyzed the immunoprecipitates side-by-side in western blots using FoxM1 antibody. The immunoprecipitates obtained with the Rb-antibody contained FoxM1 that is mainly under-phosphorylated, based on its migration (Figs 3A and S3A). FoxM1 is first phosphorylated by Cdk1/Cdk2 kinases at residue T596 that serves as a primer for phosphorylation by Plk1 at S715 and S724^42^,^43^. The phosphorylation by Plk1 is sufficient to activate the transcriptional stimulatory function of FoxM1. Therefore, to specifically investigate the Plk1-phosphorylation, we generated phospho-specific antibody against the Plk1 phosphorylation-sites (referred to as P3-ab). The P3-antibody failed to detect a mutant FoxM1 protein in which the two Plk1-phophorylation sites are mutated to alanine (Fig. S2B), confirming that the P3-ab is specific for the Plk1 phosphorylation sites. The P3-ab was validated also using knockdown experiments (Fig. S2C). We employed the P3-ab to investigate the FoxM1/Rb interaction. Interestingly, the P3-ab failed to detect any FoxM1 in the immunoprecipitates obtained with Rb-ab (Fig. 3B). The same blot when probed with the pan-FoxM1 antibody, after stripping the blot, detected FoxM1. CBP is a transcriptional co-activator of FoxM1, and it binds to Plk1-phosphorylated form of FoxM1 with greater efficiency^43^. Consistent with that, the P3-ab detected FoxM1 in the immunoprecipitates obtained with the CBP-ab (Fig. 3B). Moreover, the P3-ab co-immunoprecipitated CBP, but not Rb (Fig. 3C). Together, these observations suggest that Plk1 phosphorylation of FoxM1, which is critical for binding to CBP^43^, inhibits binding to Rb. Plk1-mediated regulation of the FoxM1/Rb interaction was confirmed further in experiments in which cells were treated with a specific inhibitor of Plk1 (BI 2536, 100 nM for 24 h). Inhibition of Plk1 was confirmed by assaying for cleaved PARP (Fig. S2D). Inhibition of Plk1 caused increase in interactions with both Rb and DNMT3b (Figs 3D and S2E). A relatively short exposure of the blot is shown in Fig. 3D to show the extent of difference in binding in the presence of the Plk1-inhibitor. Moreover, expression of kinase-active Plk1, but not a kinase-dead Plk1 mutant, reduced the interaction of FoxM1 with Rb in co-immunoprecepitation experiments (Figs 3E and S3F).

**Figure 4:**
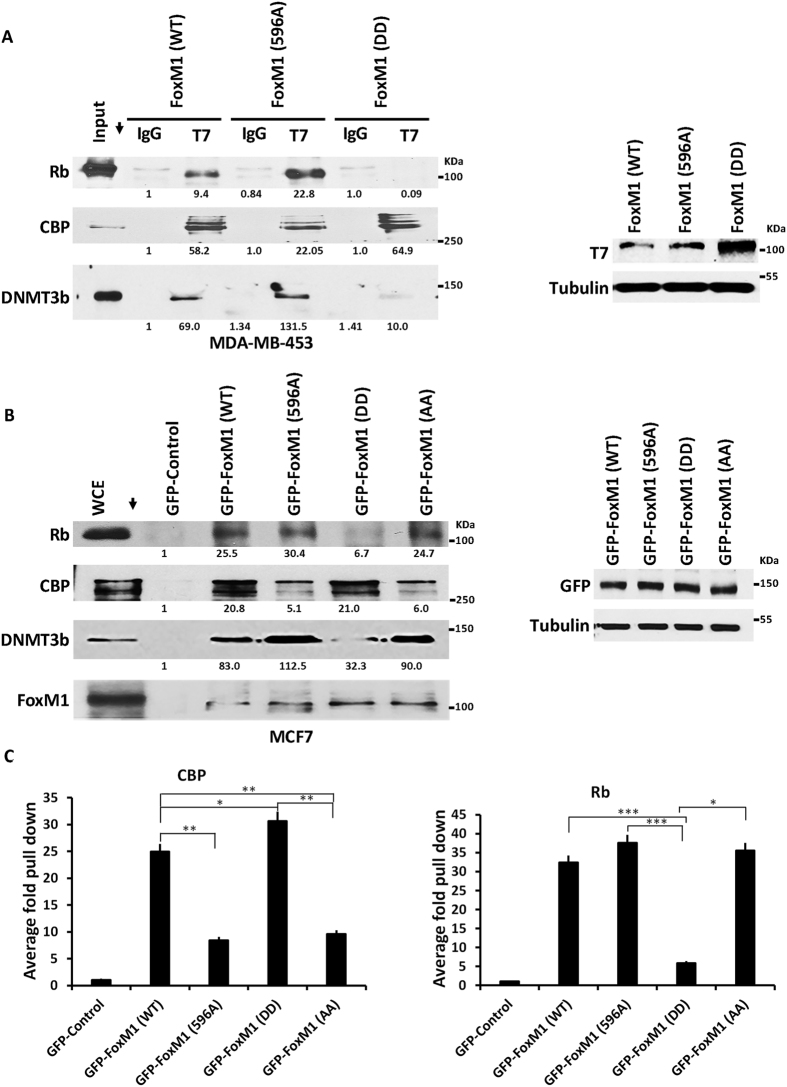
Plk1 Phosphorylation determines the binding partners of FoxM1b. (**A**) Phosphorylation of FoxM1 inhibits the interaction with Rb. MDA-MB-453 cells were transiently transfected with T7 tagged FoxM1 wild type (WT), T7 tagged phosphodefective mutant (T596A), and T7 tagged phosphomimetic mutant of S715 and S724 (FoxM1-DD). Cell lysates (800 ug) from each transfection were immunoprecipitated with a monoclonal T7 antibody. Western analysis of the upper and lower portion of the same blot was performed with CBP and Rb antibody respectively (middle and upper panel of Fig. 4A). Similar immunoprecipitations were performed in parallel to identify the interactions of these constructs with DNMT3b using a monoclonal antibody. Expression of T7-tagged wild type and phosphomutant proteins was shown in right panel. (**B**) Interactions of wild type and mutant FoxM1 with Rb in MCF7 cells. We performed the similar experiment in MCF7 cells as described in Fig. 4A with GFP-WT-FoxM1 and GFP- FoxM1 mutants. Cell lysates were immunoprecipitated with GFP antibody and the immunoprecipitates were assayed for the presence of Rb, CBP, DNMT3b, and FoxM1 after western transfer. Expression of GFP constructs was shown in right panel. (**C**) Quantifications of the Rb and CBP binding by the wild type and FoxM1-mutants was done using image J (p-values represented as *≤0.05; **≤0.01; ***≤0.001).

To further investigate the impact of Plk1 phosphorylation of FoxM1, we employed phospho-defective and phospho-mimetic mutants of FoxM1b. Mutation of T596 to alanine (T596A) was shown to inhibit Plk1 phosphorylation at S715 and S724^43^. Also, we generated S715A, S724A (AA) as Plk1 sites phospho-defective mutant, and S715D, S724D (DD) as Plk1 sites phospho-mimetic mutant. Epitope-tagged FoxM1b expression vectors were used to distinguish from the interactions of the endogenous FoxM1. GFP-tagged FoxM1b-mutants or T7-tagged mutants were analyzed for binding to Rb, CBP and DNMT3b in MDA-MB-453 (Fig. 4A) and MCF7 cells (Fig. 4B). Consistent with the experiments in Fig. 3, we observed that the phospho-defective mutants of FoxM1 maintain interactions with Rb (Fig. 4A,B). Quantifications of the binding data in MCF7 cells from 3 independent experiments (Figs 4B and S3D) are shown in Fig. 4C. Clearly, the phospho-mimetic mutant of FoxM1 is impaired in binding to Rb (Fig. 4). The opposite is true for interaction with CBP. The phospho-defective mutants are deficient in binding to CBP, but the phospho-mimetic mutant bound CBP as efficiently as the wild type, if not better (Fig. 4). The Rb-family protein p130 exhibited similar binding pattern with the mutant FoxM1 as Rb, whereas p107 appeared to retain some binding with the phospho-mimetic mutant of FoxM1 (Fig. S4). These observations on differential interactions of FoxM1 with Rb and CBP as a function of Plk1 phosphorylation suggest that Plk1 phosphorylation of FoxM1 functions as a switch that inhibits interaction with Rb or Rb-family proteins while promotes interaction with CBP. Interestingly, we observed that Plk1 phospho-mimetic mutant is deficient also in binding to DNMT3b (Figs 4 and S3B). That is somewhat surprising given that the DNMT3b binds to the N-terminal region in FoxM1. However, there is evidence for interactions between the N- and C-terminal domains of FoxM1b that is regulated by phosphorylation^50^,^51^. Therefore, in the context of the full-length protein, it is not unexpected that phosphorylation of FoxM1b at the Plk1-sites would regulate its interaction with DNMT3b.

### Bacterially produced Plk1-site phospho-mimetic mutant of FoxM1b binds to the KIX domain of CBP, but fails to interact with recombinant Rb

**Figure 5:**
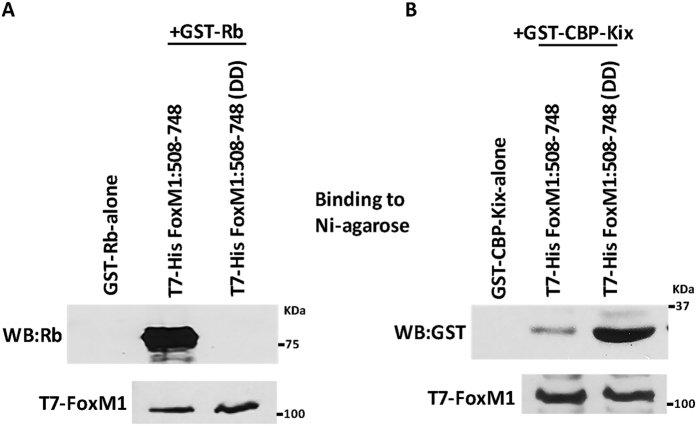
A Plk1-site phospho-mimetic mutant of FoxM1b fails to bind Rb *in vitro*. T7-His tagged C-terminal FoxM1 (residues 508–748), Plk1-site phospho-mimetic DD mutant, and GST-Rb (residues 379–928) were all expressed separately in *E. coli*. The bacterial lysates of the wild type or DD mutant FoxM1 were mixed with the lysates containing either GST-Rb or GST-CBP-KIX and then were allowed to bind Ni-agarose column. The eluted proteins, after extensive washing of the column, were assayed for the presence of Rb and CBP by western blotting (**A** and **B**). The left lane in each of the panels indicates the absence of Rb or CBP-KIX in the column elute when GST-Rb or CBP-KIX were passed through the Ni column in absence of FoxM1.

To further investigate the impact of Plk1 phosphorylation on direct interactions of FoxM1 and Rb, we generated T7-His tagged FoxM1b containing C-terminal residues 508 to 748 and a mutant version harboring DD residues at the Plk1 phosphorylation sites. The wild type and the DD mutant were expressed in *E. coli*. GST-Rb (residues 379–928) also was expressed in *E. coli*. The bacterial lysates of the wild type or DD mutant FoxM1b were mixed with the lysates containing GST-Rb first and then were allowed to bind Ni-agarose column, and after extensive washing the bound proteins were eluted and assayed for the presence of Rb by western blots. There was a significant difference in binding of Rb to the wild-type version of FoxM1b compared to the DD mutant (Fig. 5A). These observations confirm that FoxM1b directly binds to Rb, and that Plk1-mediated phosphorylation at the serine residues 715 and 724 inhibits that direct binding. Because FoxM1b binds to CBP in a phosphorylation-dependent manner in a way similar to CREB, we investigate whether the KIX domain in CBP, which binds to phosphorylated-CREB^52^, is involved in binding to FoxM1. We carried out the binding experiment using the same strategy as in for Rb (Fig. 5A), except that in place of GST-Rb we used bacterially produced GST-CBP-KIX. As shown in Fig. 5B, the Plk1-site phospho-mimetic mutant of FoxM1, which did not bind GST-Rb, bound GST-CBP-KIX. The Plk1-site phospho-mimetic mutant of FoxM1 bound to GST-CBP-KIX much more strongly than the bacterially produced wild type version of the C-terminal FoxM1 (508–748). These observations further confirm that the Plk1-phosphorylation sites in FoxM1 serve as a switch for the interactions of FoxM1 with Rb and CBP.

### Plk1 phosphorylation converts a repressor FoxM1b to an activator FoxM1b

**Figure 6:**
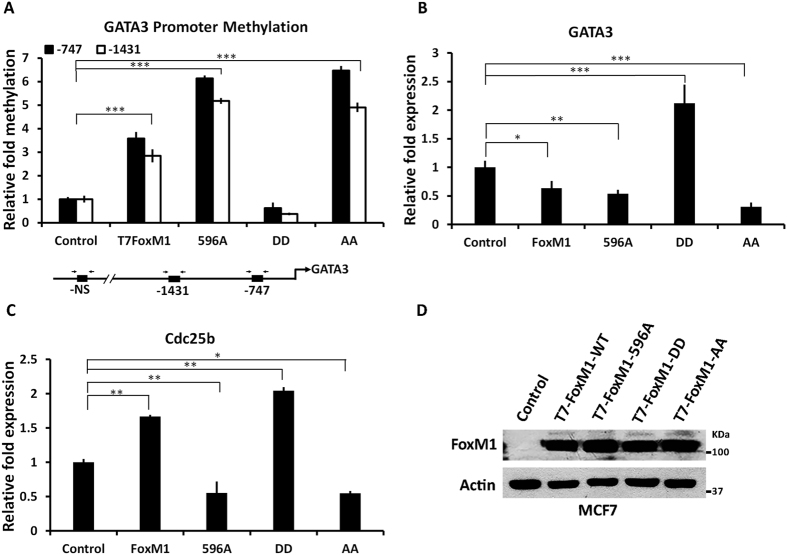
Plk1 phosphorylation regulates FoxM1b’s ability to methylate and repress GATA3 promoter. Cells were transfected with empty vector control, WT-T7-FoxM1, T7-FoxM1 (596 A), T7-FoxM1 (DD) and T7-FoxM1 (AA) expressing plasmids. Genomic DNA and RNA were extracted 48 h post transfection for GATA3 promoter methylation and transcripts quantification. (**A**). Lower panel shows the schematic of CpG island on GATA-3 promoter and primer design. In the upper panel, genomic-DNAs were isolated and C/T conversion was performed to check the promoter methylation that was quantified using the real time PCR. (**B**) Relative mRNA expression level of GATA3 in WT-FoxM1 or FoxM1 phospho-mutants expressing cells. (**C**) Relative mRNA expression level of cdc25b in the presence of WT-FoxM1 or its phospho-mutants. (**D**) Western blot showing expression of the wild type and mutant FoxM1 proteins. Statistical analysis was done using graph pad prism unpaired t test and p-values represented as *≤0.05; **≤0.01; ***≤0.001.

Previously, we showed that a FoxM1/Rb/DNMT3b complex binds to the GATA3 promoter to induce methylation at the CpG islands and repress expression of GATA3. Since the Plk1-site phospho-mimetic mutant of FoxM1 leads to a loss of the interaction of FoxM1 with Rb and DNMT3b, we predicted the mutant would be deficient in inducing GATA3 promoter CpG methylation. To test that idea we analyzed the phospho-mutants and wild type FoxM1 for their ability to induce methylation of the GATA3 promoter. Methylation-specific PCR was employed to quantify the extent of methylation at two distinct CpG islands in the GATA3 promoter. The results are plotted as fold change compared to methylation at an unspecific site. Clearly, the wild type and the phospho-defective FoxM1 caused an increase in CpG methylation, whereas the phospho-mimetic mutant failed to induce any CpG methylation (Fig. 6A).

Plk1 phosphorylation of FoxM1 is critical for its transcriptional stimulatory activity^42^,^43^. It enhances the interaction with the transcriptional co-activator protein CBP^43^. Based on the observations that Plk1 phosphorylation blocks the interaction with Rb/DNMT3b and that it inhibits methylation of the CpG-island, we predicted that it would block the transcription repression function of FoxM1. Therefore, we analyzed the Plk1 phospho-defective and phospho-mimetic mutants of FoxM1 for their effects on GATA3 expression in MCF7 cells. The phospho-defective mutants, like the wild type FoxM1, inhibited expression of GATA3, whereas the Plk1-site phospho-mimetic mutant failed to repress transcription instead there was some activation of transcription (Fig. 6B). It is noteworthy that in the same RNA preparation we did not see any repression of the FoxM1-stimulated gene Cdc25b (Fig. 6C). The phospho-defective mutants, however, inhibited expression of Cdc25b most likely by competing with the endogenous FoxM1. The effects were not a consequence of difference in expression (Fig. 6D).

**Figure 7:**
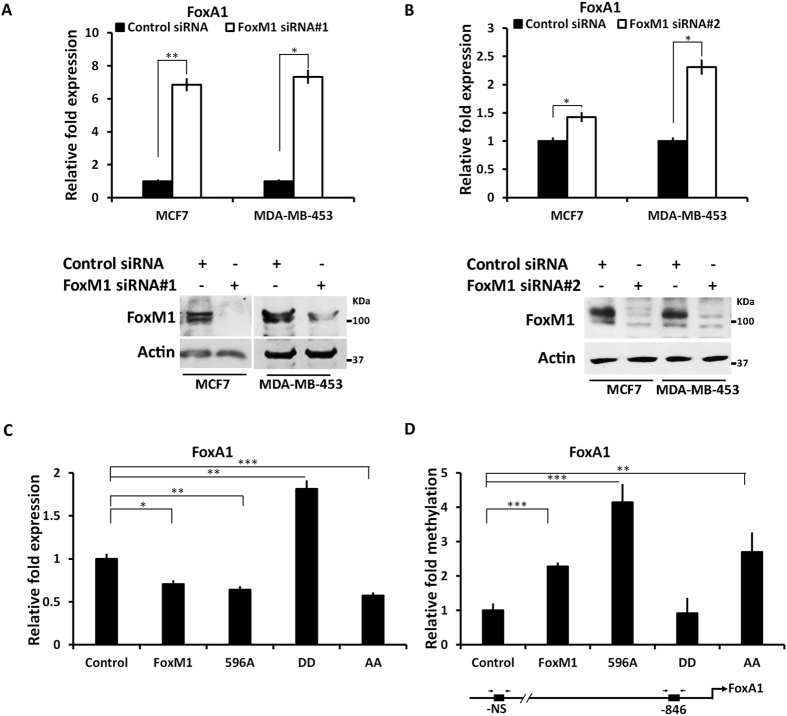
FoxM1b methylates and represses FoxA1 in MCF7 cells. (**A** and **B**) MCF7 cells and MDA-MB-453 cells were transfected with Control siRNA or two different FoxM1siRNA and 72hr post transfected cells were harvested and RNA was used to quantify the FoxA1 transcripts using qRT- PCR. Silencing of FoxM1 expression in two cell lines was verified by western blots (lower panel). (**C**) Cells were transfected with empty vector control, WT-T7-FoxM1, T7-FoxM1 (596 A), T7-FoxM1 (DD) and T7-FoxM1 (AA) expressing plasmids. RNA was extracted 48hr post transfection for FoxA1 transcripts and quantification was performed by qRT-PCR. (**D**) genomic-DNAs from the transfected cells were isolated and C/T conversion was performed to check the promoter methylation that was quantified using the real time PCR. Statistical analysis was done using Graph pad prism unpaired t test and p-values represented as *≤0.05; **≤0.01; ***≤0.001.

Interestingly, we observed that FoxM1 regulates expression of the luminal transcription factor FoxA1. Depletion of FoxM1, using two different FoxM1-siRNA in MCF7 or MDA-MB-453 cells, resulted in increased expression of FoxA1 (Fig. 7A and B). Moreover, expression of FoxM1 leads to repression of FoxA1. FoxM1 binds to the FoxA1 promoter (Fig. S5). We show that, as in the case of GATA3, the phospho-defective mutants and wild type FoxM1 repress FoxA1 expression whereas the Plk1-site phospho-mimetic mutant had no inhibitory effect instead we observed some stimulation of transcription (Fig. 7C)). Further, the phospho-mimetic mutant failed to increase methylation of the CpG-islands in the FoxA1 promoter (Fig. 7D). Together these observations suggest that the Plk1 phosphorylation sites in FoxM1 play critical roles in converting a repressor complex of FoxM1 to an activator complex.

## Discussion

The work described here is significant in several ways. First, we establish that Plk1 phosphorylation of FoxM1 regulates its interaction with Rb. And that Plk1 phosphorylation of FoxM1 impairs its ability to methylate CpG islands in the promoters of luminal differentiation genes GATA3 and FoxA1, blocking its repression function. The results identify a new molecular link between cell cycle regulators and regulation of differentiation genes (such as GATA3 and FoxA1) that offers insights also into how over-expression of FoxM1 might be playing a causal role in maintaining poorly differentiated state of cancer cells.

Plk1 is a mitotic kinase and is expressed at high levels mainly during the G2/M phases. Its expression is stimulated by FoxM1^43^. That is consistent with the observation that FoxM1 is quantitatively phosphorylated at the G2/M phases^43^. The under-phosphorylated form of FoxM1 is detected mainly in G1 phase. Also, the Rb family members are more active in the G1 phase. Therefore, the FoxM1/Rb/DNMT3b complex is expected to be most abundant in G1 phase to repress GATA3 expression. As cells progress through S and G2/M,,the level of that repressor complex is expected to diminish through phosphorylations by Cdk2/Cdk1 and by Plk1, resulting in phosphorylated FoxM1 that binds to CBP and functions mainly as an activator of the genes required for S and G2/M progression. In the mammary gland, FoxM1 is expressed at high levels in the mammary stem cells and the luminal progenitor cells, and its expression is greatly reduced in the differentiated luminal cells^1^. Consistent with that expression pattern, FoxM1 regulates differentiation of the mammary stem and the progenitor cells^1^. Drawing parallels from what is known during differentiation of the human embryonic stem cells (hESC)^53^, it is likely that FoxM1 accomplishes that by inhibiting the differentiation gene GATA3 in G1 phase and stimulating genes that inhibit differentiation in the S and G2/M phase.

Studies on differentiation of hESC revealed that G1 phase is the window of opportunity for expression of the differentiation genes^53^,^54^. As those cells undergo differentiation, the length of G1 phase increases, also the chromatins for the differentiation genes become primed for expression. For example, expression of the developmental genes in G1 phase is associated with increase in the levels of 5-hydroxymethylcytosine^55^. Smad2/3, which are activators of differentiation genes, are nuclear in G1 phase and are exported out of nucleus as cells progress through G1 phase by the actions of CyclinD-Cdk4/6^56^. Moreover, the Cyclin D-Cdk4/6 kinases were shown to support recruitment of co-repressor complex onto the endodermal lineage genes in hESCs while supporting recruitment of co-activator proteins onto the neuroectodermal lineage genes in late G1 phase^57^. As cells progress through S phase, the S phase checkpoint pathway counteracts differentiation^54^. It is noteworthy that the S phase checkpoint protein Chk2 stabilizes and increases the levels of FoxM1^58^. Also, the pluripotency gene Nanog is expressed mainly in the S, G2/M phases^54^, and it is one of the FoxM1 activated genes^37^. Also, Cyclin B1 that is important for maintaining pluripotency is a FoxM1-activated gene^27^,^28^. These are highly relevant to the fact that FoxM1 is over-expressed in poorly differentiated cancer cells. We think that its over-expression plays a causal role in maintaining the poorly differentiated phenotype of cancer cells by inhibiting differentiation genes (it is GATA3 and FoxA1 in breast cancer cells) in the G1 phase, and activating the pluripotency genes, as cells progress through S and G2 phases.

GATA3 is not just an activator of luminal differentiation in mammary gland it is also a potent tumor suppressor, as it inhibits metastatic progression in breast cancer^59^,^60^. Moreover, expression of GATA3 and FoxA1 in breast cancers inversely correlates with histological grades^61^. Their repression by FoxM1 is consistent with the notion that over-expression of FoxM1 drives high-grade progression. But, an involvement of Rb in the FoxM1-mediated repression of these luminal differentiation genes is counterintuitive, as it is a tumor suppressor protein. In normal mammary gland the FoxM1/Rb interaction is involved in maintaining the stem/progenitor pool by regulating their differentiation^1^. That function of the FoxM1/Rb complex in breast cancer cells would inhibit differentiation and support poorly differentiated tumor phenotype. That possibility is supported by our observations that FoxM1 inhibits expression of the differentiation genes GATA3 and FoxA1 in breast cancer cells. We think that under conditions of FoxM1 over-expression there is a gain-of-function for Rb (and Rb family proteins) in which it participates in development/maintenance of poorly differentiated breast cancer cells by inhibiting expression of differentiation genes, such as GATA3 and FoxA1.

The FoxM1/Rb complex is somewhat different from the E2F1-3/Rb complexes in that the FoxM1/Rb complex does not inhibit expression of the proliferation-associated FoxM1 target genes. For example, Cdc25b is not inhibited by the wild type FoxM1 (Fig. 6), whereas the same RNA preparations exhibit significant inhibition of GATA3. We speculate that the CpGs in the Cdc25b promoter are not accessible to the FoxM1/Rb/DNMT3b complex. The phospho-defective mutants inhibited Cdc25b expression in our experiments (Fig. 6). It is likely that they inhibited by a different mechanism, for example, competing with the endogenous FoxM1. In the case of E2F1-3, phosphorylation of Rb is sufficient to activate the transcription factor that stimulates the proliferation genes^62^. In the case of FoxM1, however, additional phosphorylation of FoxM1 by Plk1 is critical for conversion of the repressor to an activator, and that is related to a loss of interactions with the Rb family proteins as well as DNMT3b. Interestingly, DNMT3b and Rb could independently interact with the under-phosphorylated FoxM1; however, the Plk1-mediated phosphorylation of FoxM1 disrupts both interactions. The Plk1-phosphorylation sites overlap with the Rb-binding site, and *in silico* modeling of the FoxM1/Rb interaction (Supplemental Fig. S6), in which S715 of FoxM1 interacts with residues in the pocket domain of Rb, is consistent with the observation that phosphorylation S715 would inhibit the interaction. The opposite is expected for binding to the R624 residue of the KIX domain of CBP. Given the DNMT3b binds to the N-terminal region, it is likely that the loss of DNMT3b interaction is an indirect effect of FoxM1 phosphorylation by Plk1. It is noteworthy that there is evidence for an interaction between the N- and C-terminal domains of FoxM1 that is regulated by phosphorylation^51^. The Rb-binding region in FoxM1 we identified here is different from what was seen in a previous study^49^. One possibility for the apparent discrepancy is that we used FoxM1b in our assays, whereas the other study used FoxM1c isoform that contains sequences encoded by an additional exon next to the DNA-binding domain. Also, different assays were used in the two studies. We used co-immunoprecipitation experiments to analyze the FoxM1 mutants for *in vivo* binding, and bacterially produced recombinant proteins to detect *in vitro* binding. The bacterially produced proteins exhibited binding in Fig. 5 that is consistent with the predictions from co-immunoprecipitation assays is Fig. 4. The mutational analyses on FoxM1c were carried out mainly *in vitro*, using GST pull-down assays in the presence of reticulocyte lysate or total cell-extracts. It remains possible that other proteins in cell extracts or reticulocyte lysate mediated the interaction of Rb with the central regulatory domain of FoxM1c. Clearly, additional experiments comparing FoxM1b and FoxM1c isoforms will be required to explain the differences in the two studies.

We think that Plk1 phosphorylation of FoxM1b insures a total loss of binding to any available under-phosphorylated Rb or Rb-family members and at the same time weakens the interaction with DNMT3b. The loss of the Rb/DNMT3b interactions coincides with an increased interaction with CBP, which is critical for the transcriptional activation function of FoxM1b; thus, allowing a repressor function of FoxM1 in G1 phase and an activator function in the S and G2/M phases. The results thus provide insights also into how Plk1 phosphorylation coordinates the repressor and activator functions of FoxM1b as cells progress through the cell cycle.

## Material and Methods

Antibodies FoxM1, CBP, GFP, p107, and p130 were from Santa Cruz Biotechnology. Rb, GFP (used for western blots) were from Cell Signaling Technology. DNMT3b antibody was from Novas Biologicals. T7- Tag antibody was from Novagen. Alpha-tubulin antibody from Sigma; Phospho PLK antibody was generated in UIC core facility (P3). C-DNA synthesis kit and real-time PCR kit were from Bio Rad. The ECL detection kit was from Thermo Scientific. RNA μextraction reagent Triszol was from Invitrogen. QuickChange II XL site-directed mutagenesis kit was from Agilent Technologies. The Plk1 inhibitor BI 2536 was obtained from Selleckchem.

### Cell Culture

MCF7 and MDA-MB-453 cell lines were obtained from American Type Culture Collection (ATCC). MCF7 cells were cultured in DMEM, whereas MDA-MB-453 cells were cultured in RPMI 1640. Media was supplemented with 10% fetal bovine serum, 2% glutamine and 1% antibiotics (Pen/Strep).

### Plasmids and Transfections

Plasmid transfection was done either by lipofectamin (Invitrogen) or by X-tremeGene transfection reagent from Roche according to their protocols.

### Immunoprecipitation and Western blot Analysis

Cell lysate (about 800 μg) was immunoprecipitated with specific antibodies with either protein A or G-Sepharose beads. Bead-bound complexes were immunodetected after western transfer in nitrocellulose paper. Dilution for primary antibodies was 1:1000 for Rb, DNMT3b, GFP, CBP and FoxM1. For T7 and α-Tubulin, the dilution was 1:10,000. Dry milk (10%) was used both for blocking, primary and secondary antibody dilution. Horseradish peroxidase conjugated secondary antibodies were used at 1:5000 dilution. For phospho PLK antibody, antibody was diluted in 5% BSA both for primary and secondary antibody. Detection was performed using chemiluminescence.

### Real-time PCR

RNA was isolated using Trizol (Invitrogen) and cDNA synthesis was performed using reverse transcriptase (Bio Rad). cDNA was amplified using SYBR Green master mix and analyzed via iCycler software and the delta-delta Ct method. Sense and antisense primer sets are as follows: FoxM1, 5′-GCAGGCTGCACTATCAACAA-3′ and 5′-TCGAAGGCTCCTCAACCTTA; GATA-3, 5′-TGTCAGACCACCACAACCAGAC-3′ and 5′-TGGATGCCTTCCTTCTTCATAGTC-3′; GAPDH, 5′-ACACCCACTCCTCCACCTTT-3′ and 5′-TTCCTCTTGTGCTCTTGCTG; FoxA1, 5′-CAATGACTGCTTCGTCAAGG-3′ and 5′-TAGCAGCCGTTCTCGAACAT-3′; Cdc25B, 5′-TCCTCCGCTCAAAATCACTGTG-3′ and 5′-TGCTGAACTTGCCCGTCAATAG-3′. Samples for Q-PCR were analyzed in triplicate relative to the internal standard GAPDH RNA.

### Chromatin Immunoprecipitation

MDA-MB-453 cells were fixed for 10 min in 1% formaldehyde and quenched with glycine (125 nM). Sonicated cell lysate was incubated with either IgG or FoxM1 antibody and collected with protein-A and protein-G beads with salmon sperm DNA. DNA was extracted from beads and PCR amplification was done with the following primers: FoxA1 sense, 5′-GATGGTGCGTGTGTTGTTTTGAG-3′ and antisense, 5′-ACAAAGCACAGGGAAAAAGG-3′. PCR was performed for 30 cycles consisting of 1 min denaturation at 95 C, 1.5 min annealing at 62 C and 1.5 min elongation at 72 C. PCR products (269 bp) were visualized on a gel. FoxM1 primers for the verification of the silencing was as follows: FoxM1 sense: 5′-GCAGGCTGCACTATCAACAA-3′ and antisense, 5′-TCGAAGGCTCCTCAACCTTA.

### Bisulfite treatment and Quantitative methylation-specific PCR assay (qMSP)

Genomic DNA was isolated from MCF7 cells using the Wizard Genomic DNA isolation kit (Promega), according to the manufacturer’s protocol. EZ DNA methylation-gold kit (Zymo Research, Orange, CA) was used for sodium bisulfite conversion of gDNA according to the manufacturer’s recommendation. The methylated status of the promoter human GATA3 gene was then amplified with pairs of specific primers^1^ by SYBR green master mix and analyzed via iCycler software and delta-delta Ct method as mentioned earlier.

### Bacterial protein purification

Expression plasmids FoxM1b (508-748), FoxM1b (DD) mutant (508-748) were cloned into PET21 vector containing both His and T7 tags. GST-CBP-KIX expression plasmid was obtained from the lab of Dr. M.R. Montminy (The Salk Institute, La Jolla, CA 92037). Cloned plasmids were first transformed into BL21 and a colony grown on each LB/Amp plate was grown in LB in presence of IPTG for optimum expression. Ni-NTA agarose columns were prepared and equilibrated by washing with 10 mM imidazole. Sonicated bacterial extract containing GST-RB or GST-CBP-KIX was mixed either with WT FoxM1 or FoxM1^63^ extract and was added in two separate Ni columns. The columns were washed 10 times with 25 mM imidazole in PBS (pH 7.5). The bound proteins were eluted from each column with 250 mM imidazole. Equal volume of aliquot from each column was loaded in 8% polyacrylamide gel, transferred to nitrocellulose, and immune-detected for Rb binding with a mono-specific Rb antibody or GST-ab to detect GST-CBP-KIX.

### Statistical Analysis

Experiments were repeated independently several time and reproduced at least three times and values were expressed as means ± standard deviation (SD). We used Microsoft Excel to calculate standard deviations and statistically significant differences between samples using the student t test. Either exact *P*-value or a *P*-value of 0.05 or less was considered significant and has been used.

## Additional Information

**How to cite this article**: Mukhopadhyay, N. K. *et al*. Plk1 Regulates the Repressor Function of FoxM1b by inhibiting its Interaction with the Retinoblastoma Protein. *Sci. Rep.* **7**, 46017; doi: 10.1038/srep46017 (2017).

**Publisher's note:** Springer Nature remains neutral with regard to jurisdictional claims in published maps and institutional affiliations.

## Supplementary Material

Supplementary Information
