# Author Correction: Plk1 Regulates the Repressor Function of FoxM1b by inhibiting its Interaction with the Retinoblastoma Protein

**DOI:** 10.1038/s41598-018-34071-w

**Published:** 2018-10-29

**Authors:** Nishit K. Mukhopadhyay, Vaibhav Chand, Akshay Pandey, Dragana Kopanja, Janai R. Carr, Yi-Ju Chen, Xiubei Liao, Pradip Raychaudhuri

**Affiliations:** 1 Department of Biochemistry and Molecular Genetics (M/C 669), University of Illinois, College of Medicine, 900 S, USA Ashland Ave., Chicago, IL 60607 USA; 2 Department of Hematology/Oncology, University of California, Los Angeles, CA USA; 3 Abramson Family Cancer Research Institute, University of Pennsylvania, Philadelphia, PA 19104 USA; 4 Jesse Brown VA Medical Center, 820 S. Damen Ave., Chicago, IL 60612 USA

Correction to: *Scientific Reports* 10.1038/srep46017, published online 07 April 2017

This Article contains an error in Figure 5, where the T7-FoxM1 panels have been erroneously written as 100 and not 25. The correct Figure 5 appears below as Figure 1.

**Figure 1:**
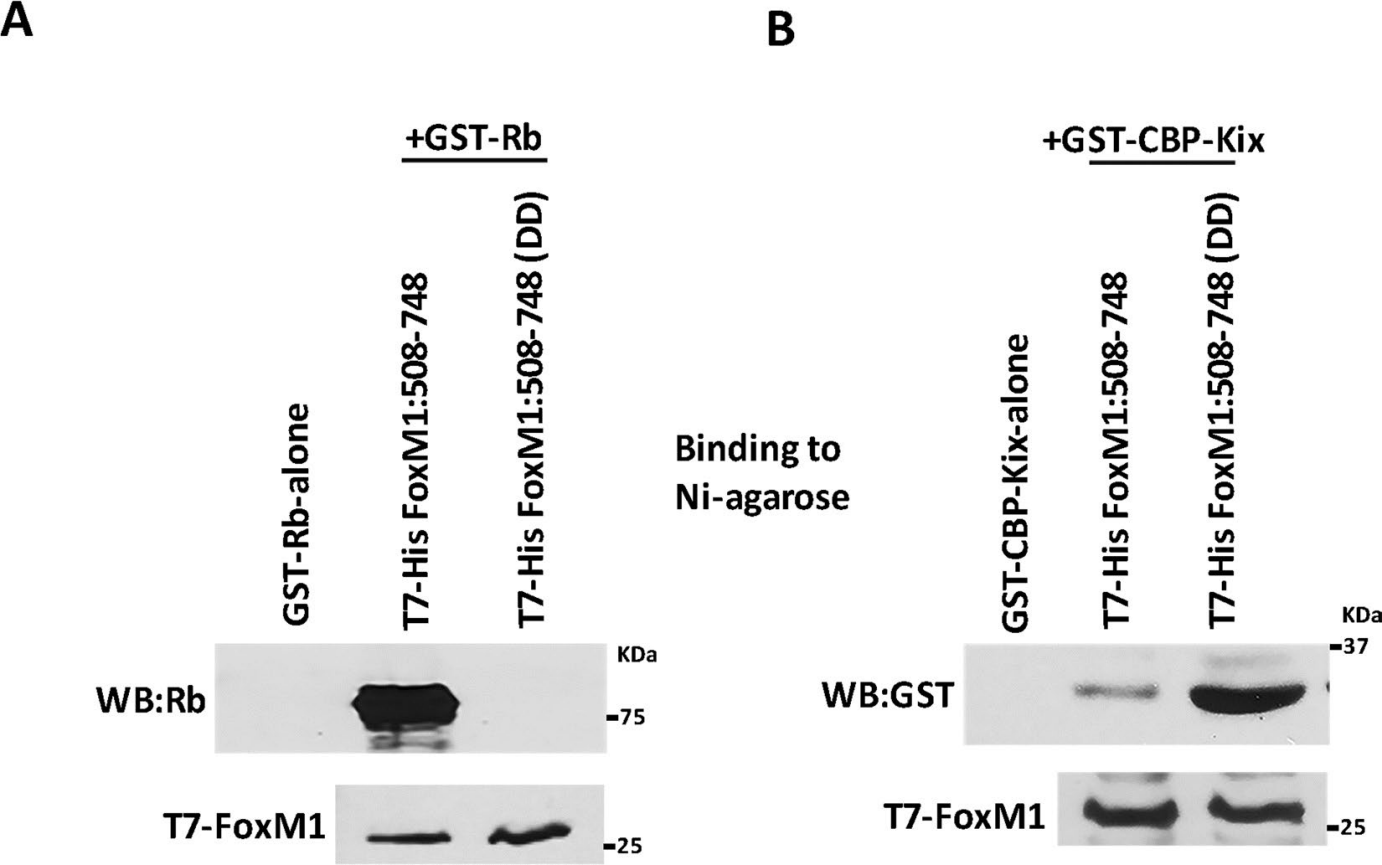
A Plk1-site phospho-mimetic mutant of FoxM1b fails to bind Rb *in vitro*. T7-His tagged C-terminal FoxM1 (residues 508–748), Plk1-site phospho-mimetic DD mutant, and GST-Rb (residues 379–928) were all expressed separately in *E. coli*. The bacterial lysates of the wild type or DD mutant FoxM1 were mixed with the lysates containing either GST-Rb or GST-CBP-KIX and then were allowed to bind Ni-agarose column. The eluted proteins, after extensive washing of the column, were assayed for the presence of Rb and CBP by western blotting (**A** and **B**). The left lane in each of the panels indicates the absence of Rb or CBP-KIX in the column elute when GST-Rb or CBP-KIX were passed through the Ni column in absence of FoxM1.

