# Supplementary material for: Plk1 Regulates the Repressor Function of FoxM1b by inhibiting its Interaction with the Retinoblastoma Protein
Source: Sci Rep. 2017 Apr 7;7:46017. doi: 10.1038/srep46017 (PMC5384083; doi:10.1038/srep46017)
Supplement: Supplementary Information [file srep46017-s1.pdf]

Supplementary Figures and Legends for manuscript #SREP-16-22740-T

Plk1 Regulates the Repressor Function of FoxM1b by inhibiting its Interaction with the Retinoblastoma Protein

Authors: Nishit K Mukhopadhyay (1)# Vaibhav Chand (1)# Akshay Pandey (1), Dragana Kopanja (1), Janai R. Carr (2), Yi-Ju Chen (3), Xiubei Liao (1) and Pradip Raychaudhuri (1, 4)*

Addresses: (1) Department of Biochemistry and Molecular Genetics (M/C 669), University of Illinois, College of Medicine, 900 S. Ashland Ave., Chicago, IL-60607. (2) Department of Hematology/Oncology, University of California, Los Angeles, CA. (3) Abramson Family Cancer Research Institute, University of Pennsylvania, Philadelphia, PA 19104. (4) Jesse Brown VA Medical Center, 820 S. Damen Ave., Chicago, IL-60612.

# These two authors made equal contribution.

*Corresponding Author: Ph. # (312) 413 0255; Fax # (312) 355 3847.E-mailpradip@uic.edu

Supplemental Figure legends:

Fig S1: Expression of GFP constructs and immunoprecipitation profile for Rb binding. A, Western blot analysis of GFP-FoxM1 wild type and mutant constructs in MCF7 cells immunoblotted with mouse GFP-ab. **B,** Full-length western blot corresponding to Figure 2C.

Fig S2: Validation of P3 antibody and effect of PLK inhibitor. A, Full-length western blot corresponding to Figure 3A. **B,** A phospho-specific antibody (P3) was generated using a phospho-peptide corresponding to the Plk1-sites in FoxM1. P3 antibody fails to bind FoxM1 in which Plk1-sites are replaced by alanine. Vector, T7-FoxM1 (FL), and T7-FoxM1 (AA) mutant constructs were transiently transfected in MCF7 cells and the lysates were immunoprecipitated with T7 antibody. Western blotting analysis was done using the P3 antibody. The same blot was reproved with FoxM1 antibody to do FoxM1 blot (right panel). **C,** P3 antibody was further validated by silencing FoxM1 in MCF7 cells. Intensity of the phosphorylated FoxM1 band is significantly down in FoxM1 silenced cells after 72 hr. **D,** Effect of PLK inhibitor (BI 2536) treatment in MCF7 cells for 24 hr on PARP cleavage indicating that the inhibitor was actively inhibiting Plk1 during the treatment. E, Full length western blot corresponding to Figure 3D. F, Full-length western blot of exogenous PLK1 kinase inhibits Rb binding as in Fig 3E.

Fig S3: Full length blots for phosphomutant experiments and statistical analysis. A, The full-length version of Figure 4A is shown. Binding of Rb with wild type FoxM1 and phospho-mutants of FoxM1 in MDA-MB-453 cells. Upper portion of the same blot was probed for CBP as indicated in Figure 4A. A separate set of extracts from these cells was immunoblotted for DNMT3b antibody as indicated in upper right panel (B). **C,** Full-length blot of interactions of WT FoxM1 and phosphomutants with Rb in MCF7 cells. Same blot was probed for three different antibodies

against Rb, CBP and DNMT3b (Fig. 4B). **D,** Binding data from two independent experiments in MCF7 cells are represented here in addition to the data in Fig 4B.

Fig S4: Interactions of p107 and p130 with phospho-mutants of FoxM1. T7 tagged wild type and the phospho-mutants of FoxM1 were expressed in MDA-MB-453 cells by transient transfection. Immunoprecipitated T7 tagged proteins were western transferred and analyzed using either p107 or p130 antibody as indicated in the upper and middle panel respectively. Lower panel indicates expression of the T7-tagged FoxM1proteins.

Fig S5: FoxM1 binds to FoxA1 promoter. A, shows a schematic representation of the FoxA1 promoter. The putative FoxM1 binding sites and CpG islands are indicated. **B,** The site (-1391) upstream of transcription start site of FoxA1 shows specific binding of FoxM1 on FoxA1 promoter in MDA-MB-453 cells. **C,** Silencing of FoxM1 decreased binding of FoxM1 onto the FoxA1 promoter. **D,** PCR amplification of FoxM1 level after FoxM1 silencing.

Fig S6: Computer generated model of C-terminal FoxM1 and Rb interaction. In the L (714) SLSKILLD sequence in the C-terminus of FoxM1, ISKIL is assumed to adopt a helical structure as observed in Rb-E1A complex structure (PDB: 2R7G, ref.1). This LSLSKILLD sequence then is docked to E1A-free Rb structure (3POM) (2) using the program ZDOCK (3). This program indicates that the AB pocket of Rb is a preferred binding site of the LSLSKILLD sequence. The structure is homologous to the Rb-E1A complex structure (2R7G). This resulting complex is further energy minimized by using the program FIBERDOCK (4,5). In this energy minimized complex structure, the side chain of S715 makes contacts with the side chains of L476 and E464 of Rb. This interaction is stabilized by the side chain interactions between E464 of Rb and K718 of FoxM1. This structure indicates that the phosphorylation on S715 may disrupt the side chain interactions between L476, E464 of Rb and S715 of FoxM1. The light gray structure represent

the structure of AB pocket of Rb (3POM). Red color indicates the helical structure of the C-terminal sequence L (714) SLSKILLD of FoxM1. The blue color side chains S715 and K718 represent the interaction area with Rb.0

B, Computer generated model of C-terminal FoxM1 mutant (S715D) and the CBP-KIX domain interaction (right panel). DLSKIL (S715D) sequence is assumed as the same structure of the wild type SLSKIL sequence. The DLSKIL is docked onto KIX domain in PDB structure (2LXS) (6) using ZDOCK program. The negatively charged side-chain S715D makes contact with the positively charged side-chain R624 in the KIX domain of CBP. This interaction further stabilizes S715D FoxM1-KIX interaction. A similar contact pattern is detected in other KIX protein complexes.

Supplemental References:

1. Structure of the retinoblastoma protein bound to adenovirus e1a reveals the molecular basis for viral oncoprotein inactivation of a tumor suppressor. Liu X. Marmorstein R Genes Dev. (2007) 21 p.2711
2. Crystal structure of the unliganded retinoblastoma protein domain. Balog ER, Burke JR, Hura GL, Rubin SM, Proteins (2011) 79 p.2010
3. Pierce BG, Wiehe K, Hwang H, Kim BH, Vreven T, Weng Z. (2014) ZDOCK Server: Interactive Docking Prediction of Protein-Protein Complexes and Symmetric Multimers. Bioinformatics 30(12): 1771-3

4. E. Mashiach, R. Nussinov and H. J. Wolfson, FiberDock: Flexible induced-fit backbone refinement in molecular docking. Proteins 2009 Dec9; 78(6):1503-1519
5. E. Mashiach, R. Nussinov and H. J. Wolfson. FiberDock: a web server for flexible induced-fit backbone refinement in molecular docking. Nucleic Acids res. 2010 Jul 1;38 Suppl:W457-61
6. S. Bruschweiler , R Konrat, M Tollinger: Allosteric communication in the kix domain proceeds through dynamic repacking of the hydrophobic core. ACS Chem. Biol. (2013) July 19;8(7) 1600-10.

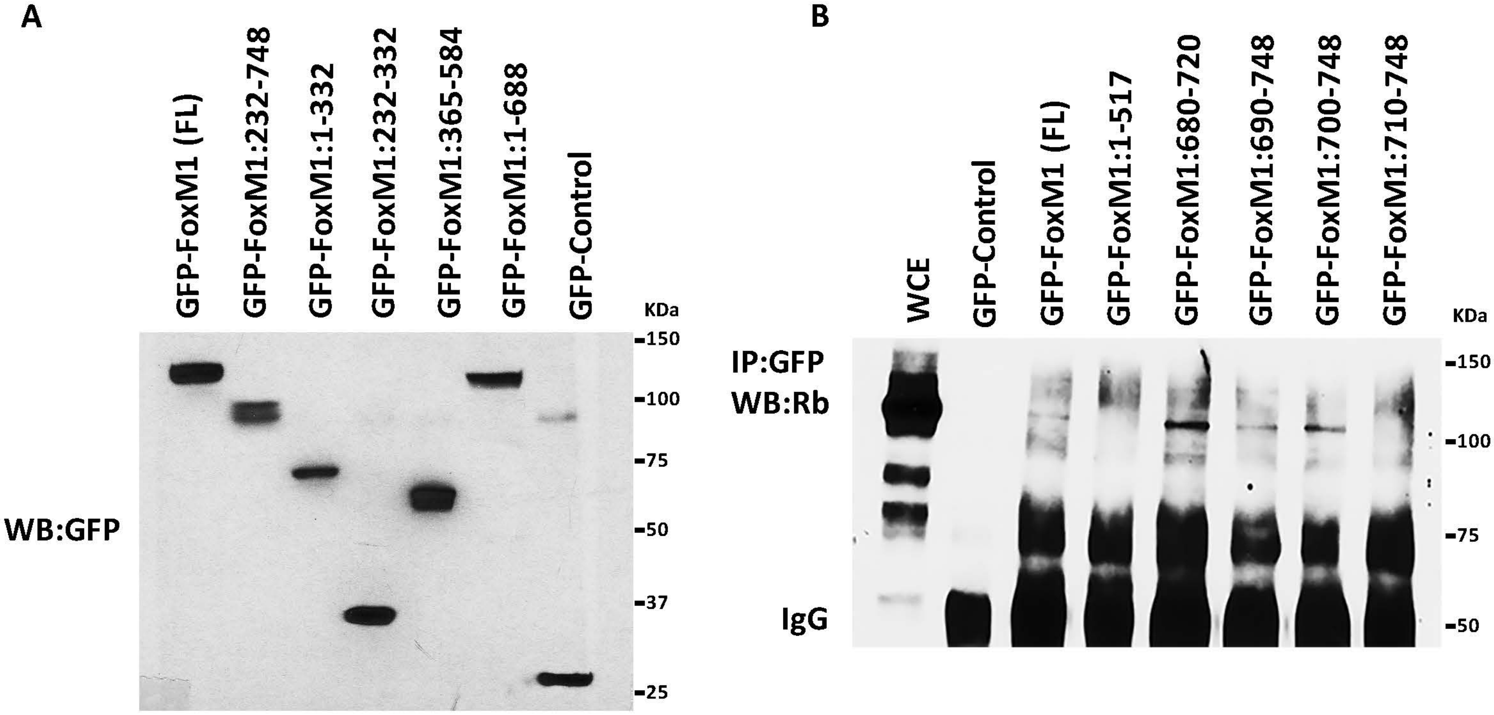
A
GFP-FoxM1 (FL)
GFP-FoxM1:232-748
GFP-FoxM1:1-332
GFP-FoxM1:232-332
GFP-FoxM1:365-584
GFP-FoxM1:1-688
GFP-Control
KDa
150
100
75
50
37
25
WB:GFP
B
WCE
GFP-Control
GFP-FoxM1 (FL)
GFP-FoxM1:1-517
GFP-FoxM1:680-720
GFP-FoxM1:690-748
GFP-FoxM1:700-748
GFP-FoxM1:710-748
KDa
150
100
75
50
IP:GFP
WB:Rb
IgG

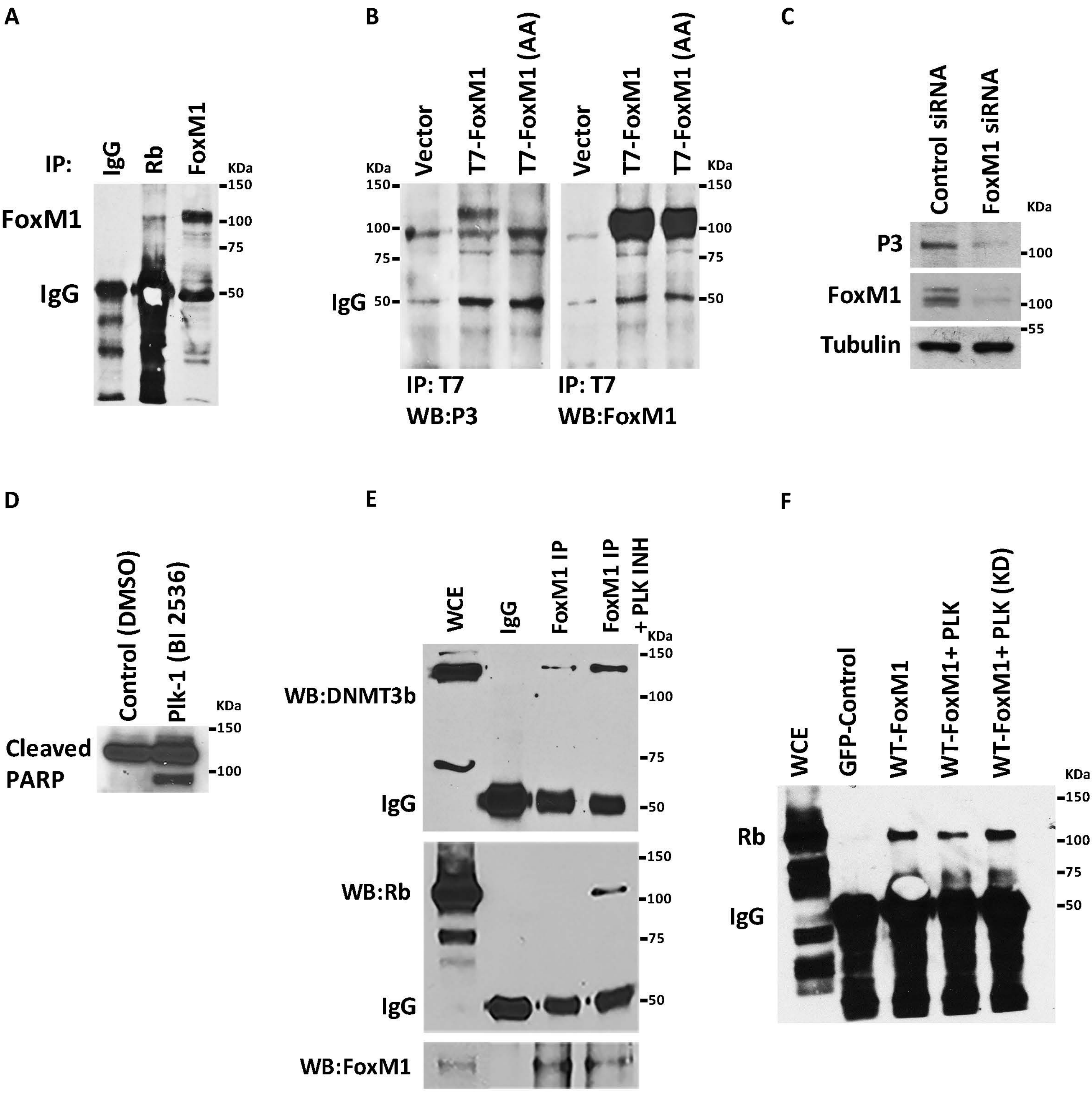
A
IP:
IgG
Rb
FoxM1
KDa
150
100
75
50
FoxM1
IgG
B
KDa
Vector
T7-FoxM1
T7-FoxM1 (AA)
Vector
T7-FoxM1
T7-FoxM1 (AA)
KDa
150
100
75
50
IgG
IP: T7
WB:P3
IP: T7
WB:FoxM1
C
Control siRNA
FoxM1 siRNA
KDa
P3
100
FoxM1
100
55
Tubulin
D
Control (DMSO)
Plk-1 (BI 2536)
KDa
150
100
Cleaved
PARP
E
WCE
IgG
FoxM1 IP
FoxM1 IP + PLK INH
KDa
150
100
75
50
WB:DNMT3b
IgG
WB:Rb
IgG
WB:FoxM1
F
WCE
GFP-Control
WT-FoxM1
WT-FoxM1+ PLK
WT-FoxM1+ PLK (KD)
KDa
150
100
75
50
Rb
IgG

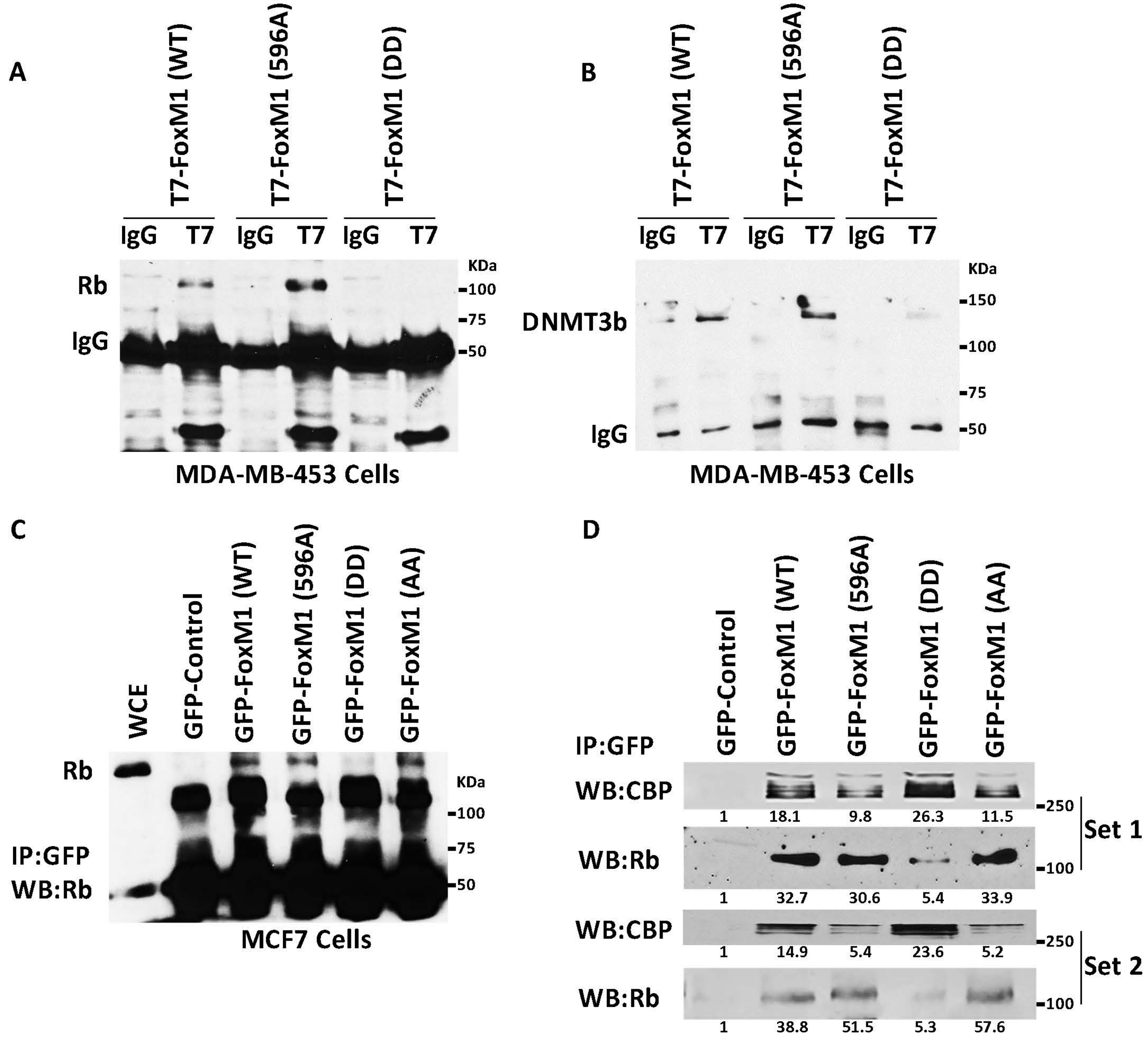
A
T7-FoxM1 (WT)
T7-FoxM1 (596A)
T7-FoxM1 (DD)
IgG T7 IgG T7 IgG T7
KDa
Rb
IgG
100
75
50
MDA-MB-453 Cells
B
T7-FoxM1 (WT)
T7-FoxM1 (596A)
T7-FoxM1 (DD)
IgG T7 IgG T7 IgG T7
KDa
DNMT3b
IgG
150
100
75
50
MDA-MB-453 Cells
C
WCE
GFP-Control
GFP-FoxM1 (WT)
GFP-FoxM1 (596A)
GFP-FoxM1 (DD)
GFP-FoxM1 (AA)
Rb
IP:GFP
WB:Rb
KDa
100
75
50
MCF7 Cells
D
GFP-Control
GFP-FoxM1 (WT)
GFP-FoxM1 (596A)
GFP-FoxM1 (DD)
GFP-FoxM1 (AA)
IP:GFP
WB:CBP
1 18.1 9.8 26.3 11.5
250
Set 1
WB:Rb
1 32.7 30.6 5.4 33.9
100
WB:CBP
1 14.9 5.4 23.6 5.2
250
Set 2
WB:Rb
1 38.8 51.5 5.3 57.6
100

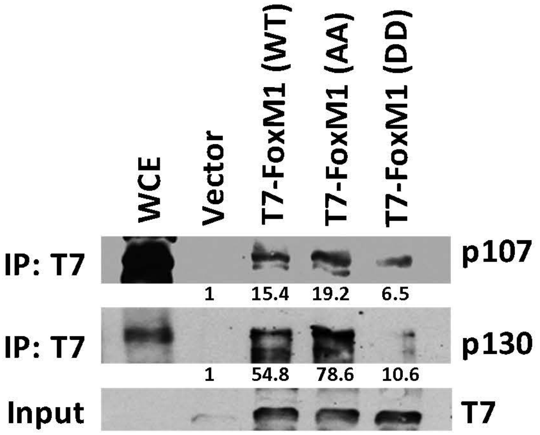
WCE
Vector
T7-FoxM1 (WT)
T7-FoxM1 (AA)
T7-FoxM1 (DD)
IP: T7
p107
1
15.4
19.2
6.5
IP: T7
p130
1
54.8
78.6
10.6
Input
T7

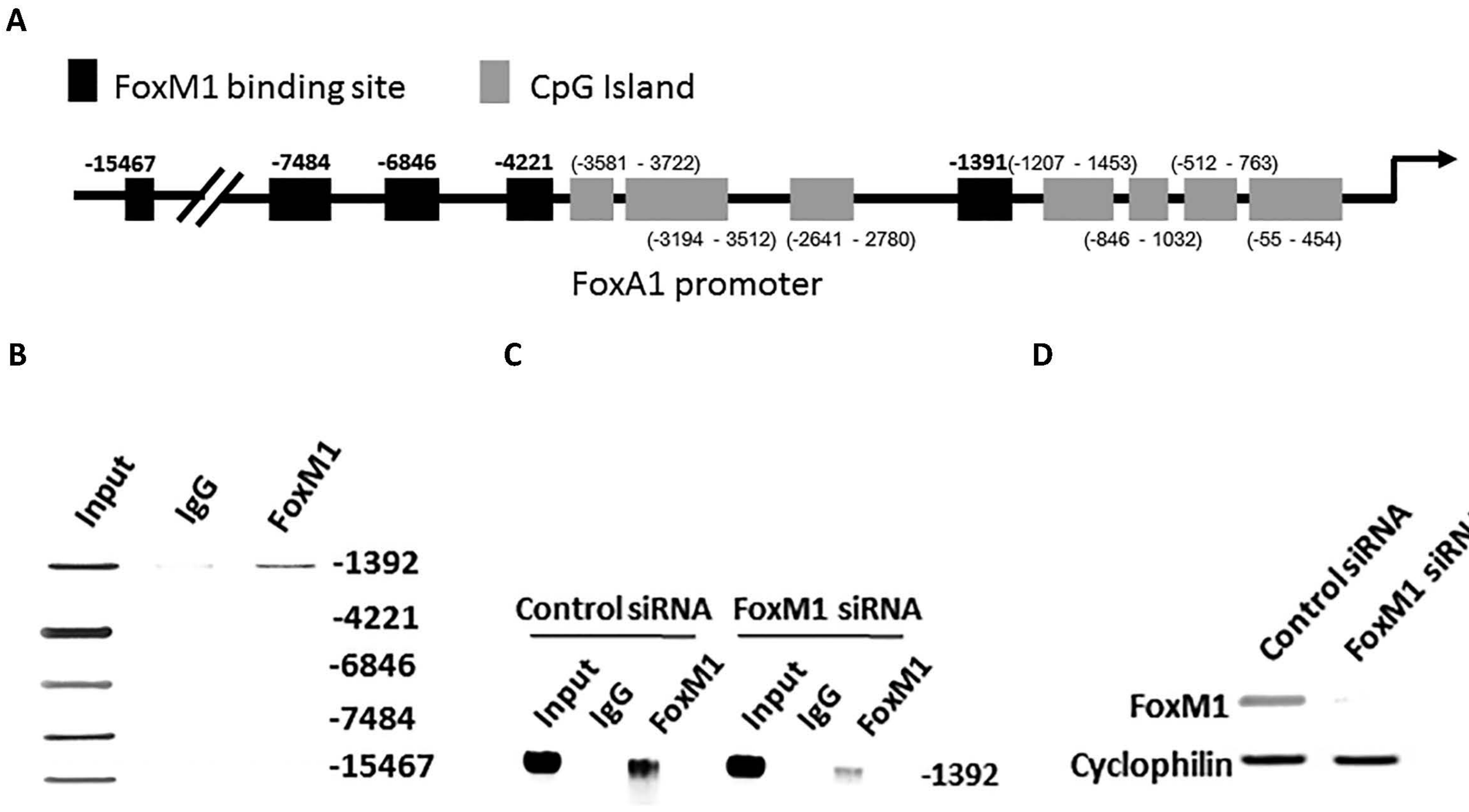
A
FoxM1 binding site
CpG Island
-15467
-7484
-6846
-4221
(-3581 - 3722)
-1391
(-1207 - 1453)
(-512 - 763)
(-3194 - 3512)
(-2641 - 2780)
(-846 - 1032)
(-55 - 454)
FoxA1 promoter
B
Input
IgG
FoxM1
-1392
-4221
-6846
-7484
-15467
C
Control siRNA
FoxM1 siRNA
Input
IgG
FoxM1
Input
IgG
FoxM1
-1392
D
Control siRNA
FoxM1 siRNA
FoxM1
Cyclophilin

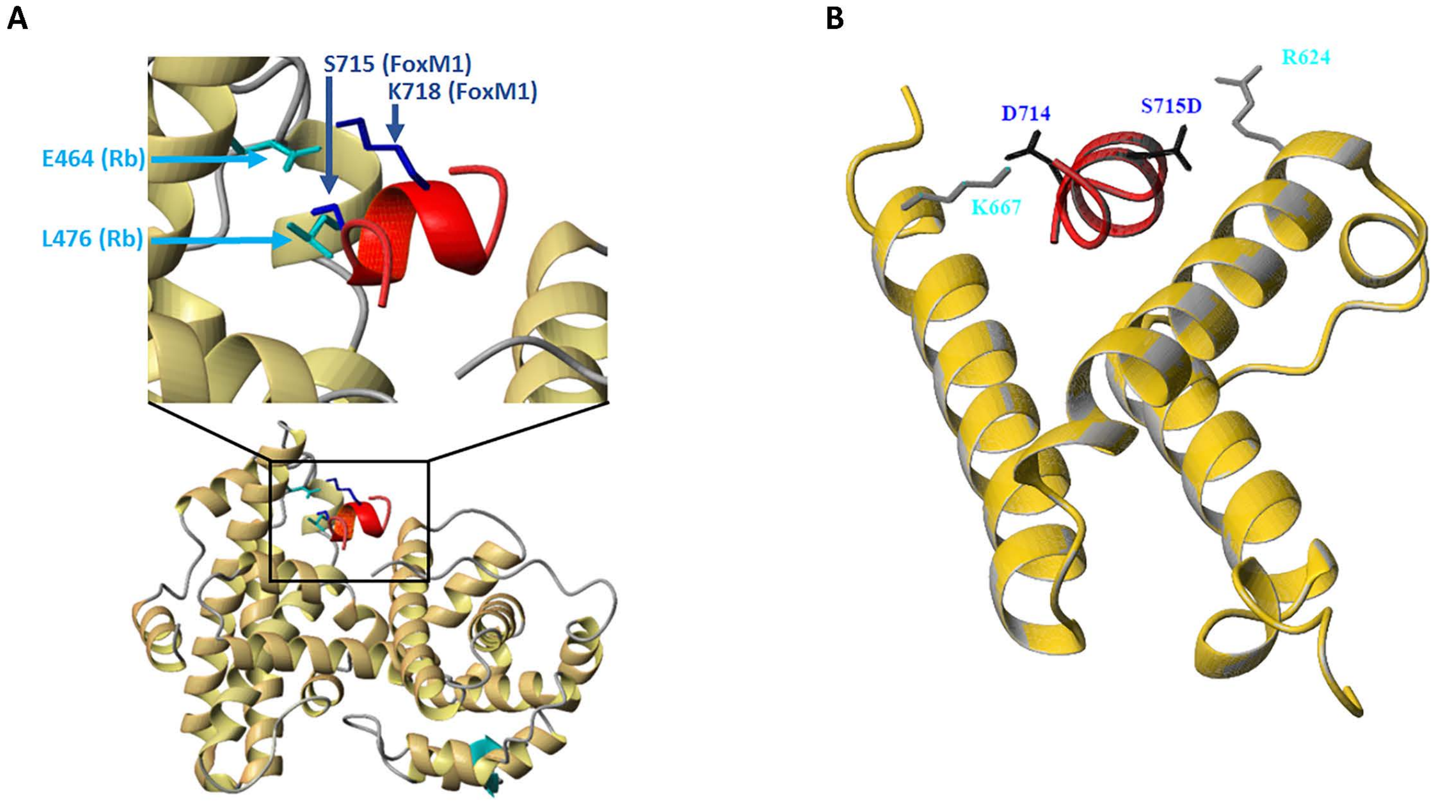
A
S715 (FoxM1)
K718 (FoxM1)
E464 (Rb)
L476 (Rb)
B
R624
D714
S715D
K667
